# The pathophysiological function of peroxisome proliferator-activated receptor-γ in lung-related diseases

**DOI:** 10.1186/1465-9921-6-102

**Published:** 2005-09-09

**Authors:** Tom Hsun-Wei Huang, Valentina Razmovski-Naumovski, Bhavani Prasad Kota, Diana Shu-Hsuan Lin, Basil D Roufogalis

**Affiliations:** 1 Faculty of Pharmacy, A15, University of Sydney, New South Wales, 2006, Australia

**Keywords:** Peroxisome proliferator-activated receptor-gamma, respiratory diseases, asthma, chronic obstructive pulmonary disease, lung cancer.

## Abstract

Research into respiratory diseases has reached a critical stage and the introduction of novel therapies is essential in combating these debilitating conditions. With the discovery of the peroxisome proliferator-activated receptor and its involvement in inflammatory responses of cardiovascular disease and diabetes, attention has turned to lung diseases and whether knowledge of this receptor can be applied to therapy of the human airways. In this article, we explore the prospect of peroxisome proliferator-activated receptor-γ as a marker and treatment focal point of lung diseases such as asthma, chronic obstructive pulmonary disorder, lung cancer and cystic fibrosis. It is anticipated that peroxisome proliferator-activated receptor-γ ligands will provide not only useful mechanistic pathway information but also a possible new wave of therapies for sufferers of chronic respiratory diseases.

## Introduction

It would be fair to say that airway diseases place a significant burden on the population in terms of health, social and economic costs. Leading the way are the chronic pulmonary disorders such as asthma and lung cancer, riddled with significant obstacles associated with their various drug treatments, including limited effectiveness, immunity and side effects. Recent studies delve into the role of inflammation in the airways and its associated army of diverse cell types including leukocytes, lymphocytes, neutrophils and eosinophils [1]. Modern treatments have focused on receptor-mediated responses in an attempt to effectively counteract a specific disease state. Recently, peroxisome proliferator-activated receptors (PPAR), in particular, PPAR-γ, have surfaced as novel immunomodulators due to their anti-inflammatory actions, most notably in cardiovascular and diabetes-related diseases [2,3]. This regulation of inflammatory responses by PPAR-γ has been extended to processes within the lung, through actions on both immune and non-immune cells [5]. Widespread clinical use of PPAR-γ agonists has provided a possible new direction in the treatment of airway inflammatory diseases through control of PPAR-γ regulated pathways [4]. This has uncovered the potential of inhaled PPAR-γ agonists in the treatment of airway inflammation via the many cellular targets in the lung such as T lymphocytes, epithelial cells and smooth muscle cells with the possibility of delivering them locally, with minimal side effects, compared to the currently available corticosteroids [5]. Current studies have allowed greater insight into the role of the receptor on the modulation of airway respiratory diseases by interaction with its agonists, 15-deoxy-Δ^12,14^-prostaglandin J2 (15D-PGJ_2_) and thiazolidinediones (TZD). This review will summarise the connections between PPAR-γ interactions with agonists and the mechanisms involved in lung cellular processes in chronic diseases such as asthma, lung cancer, cystic fibrosis and chronic obstructive pulmonary disease (COPD).

## PPARs: Background

Since the turn of the decade, the science of receptor-mediated responses has progressed rapidly, uncovering many unknown pathways of pharmaceutical drug action and, lately, targeting many diseases where conventional medicine has had limited success. The literature on the PPAR physiology is extensive. Briefly, the PPARs are a family of transcription factors belonging to the nuclear hormone receptor superfamily [6,7]. Three PPAR isoforms, designated PPAR-α (NR1C1), PPAR-β (also called PPAR-δ, FAAR, NuC1 or NR1C2) and PPAR-γ (NR1C3) have been cloned and are differentially expressed in several tissues including liver, kidney, heart and muscle. PPAR-α primarily regulates cellular lipid metabolism and modulates inflammation. PPAR-β participates in embryonic development, implantation and bone formation. PPAR-γ, which is the focus of this review, is a key factor in adipogenesis and is primarily advocated in insulin sensitivity, cell cycle regulation and cell differentiation [6]. A large proportion of PPARs actions are mediated through binding to PPAR-response elements (PPRE) on DNA. PPRE are constituents of direct repeat (DR) hexameric sequences (AGGTCA), which are separated by one or two nucleotides (DR-1 and DR-2 element). Distinct areas such as the DNA binding and the ligand-independent transactivation domains have been identified and these influence the transduction of the PPAR-induced response [8]. PPARs **heterodimerise **with the 9-cis-retinoic acid receptors (RXR) and the resultant heterodimer subsequently binds to PPRE with the recruitment of cofactors. PPARs regulate numerous genes through ligand-dependent transcriptional activation and repression. This conformational interaction has a profound affect on numerous cellular processes, including lipid metabolism, glucose homeostasis, cell cycle progression, cell differentiation, inflammation and extracellular matrix remodelling [9]. The localisation of a ligand to the ligand-binding domain results in a conformational change of the receptor, thereby allowing transactivation of the appropriate genes [6]. The natural prostaglandin D2 metabolite, 15D-PGJ_2 _and synthetic anti-diabetic TZDs are principal ligands of PPAR-γ and will be the focus of the review.

### Expression and physiological role of PPAR-γ in lung

#### Expression

**Figure 1:**
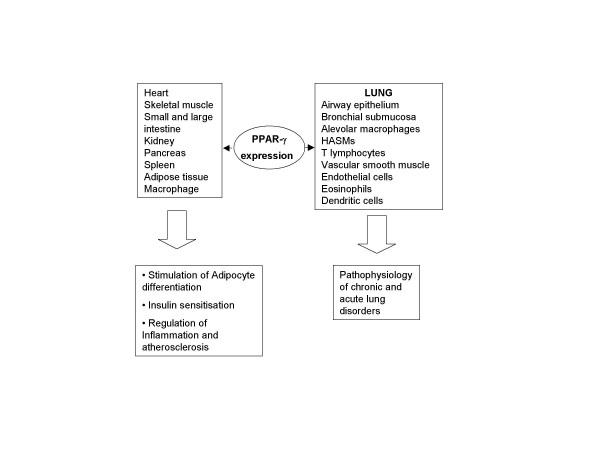
**Expression of PPAR-γ in various tissues and its role in lung and other organs. **PPAR-γ ligands implicated in the treatment of chronic inflammatory disorders in lung. Activation of PPAR-γ in heart, intestine, kidney, skeletal muscle, pancreas, macrophages and adipose tissue results in energy homeostasis and this effect also found to be crucial in the pathophysiology of different disorders. Please refer text for more information.

Historically, the discovery of PPAR-α led to the subsequent identification of other isoforms such as PPAR β/δ and PPAR-γ [10]. The PPAR-γ gene contains three promoters that yield three sub-isoforms, namely, PPAR-γ_1_, PPAR-γ_2 _[11] and PPAR-γ_3 _[12]. A comparison of the tissue-distribution of PPAR-γ transcripts among different species illustrates the presence of PPAR-γ_1 _in a broad spectrum of tissues such as heart, skeletal muscle, small and large intestine, kidney, pancreas and spleen, whereas PPAR-γ_2 _is restricted to adipose tissue [6]. Structurally, PPAR-γ_2 _contains an additional 30 amino acids at the N-terminal end relative to PPAR-γ_1_. PPAR-γ_3 _is abundant in macrophages, the large intestine and white adipose tissue [12]. Specific to the distribution of PPAR-γ in lung, the expression of PPAR-γ_1 _was exhibited at relatively high levels in bovine lung compared to PPAR-γ_2_. The cellular expression profile of PPAR-γ in pulmonary tissue has not been well characterised, but studies have uncovered abundant expression of PPAR-γ in airway epithelium [13], in bronchial submucosa [14], in mononuclear phagocytes such as human alveolar macrophages (AM) [3], human T lymphocytes [2], in two different human bronchial epithelial cells, NL20 and BEAS [15] and human airway smooth muscle (HASM) cells [2,16]. In HASM cells, PPAR-α but not PPAR-β was expressed [47]. Primary normal human bronchial epithelial cells and human lung epithelial cell lines BEAS 2B, A549 and NCI-H292 all express PPAR-γ and PPAR-β, but not PPAR-α [28]. Both PPAR-α and PPAR-γ are expressed by eosinophils [29]. Mice, rat and human lung models have been pivotal to the greater understanding of the mechanistic pathways related to PPAR-γ and the various lung diseases (Figure 1).

#### Physiology

**Figure 2:**
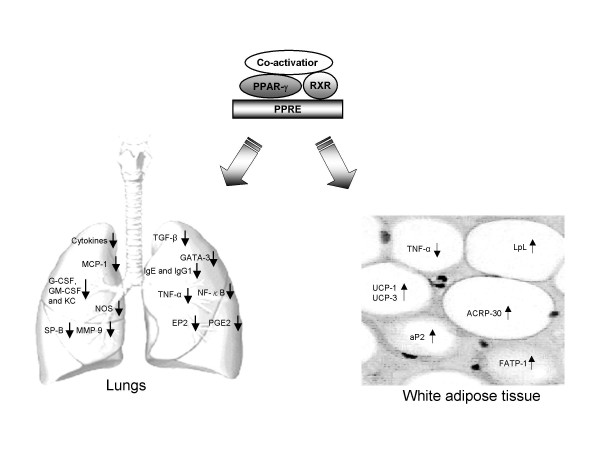
**Activation of PPAR-γ by endogenous (15D-PGJ2) and exogenous (TZDs) ligands results in transcription of wide array of genes that can control pathogenesis of acute and chronic disorders in various tissues of lungs. **Please refer text for more information. Abbreviations: 15D-PGJ2: 15-deoxy-Δ^12,14^-prostaglandin J2, Cpla2: cytosolic phospholipase A_2_, TZDs: Thiozolidinediones NSAIDs: Non-steroidal anti-inflammatory drugs MCP:1monocyte chemoattractant protein, G-CSF: granulocyte-colony-stimulating factor, GM-CSF:granulocyte-macrophage-colony-stimulating factor, KC: keratinocyte-derived chemokine, NOS: Nitric oxide synthases, SP-B: surfactant proteins-B, MMP-9: matrix metalloproteinase 9, TGF-β: Transforming growth factor-β, IgE and IgG1: Immunoglubulin E and Immuno globulin G1, NF-κB: Nuclear factor-κB, EP2: Prostaglandin E2 receptor, PGE2: Prostaglandin E2, aP2: Adipocyte fatty acid binding protein, UCP 1&3: Uncoupling proteins 1 & 3, Acrp30: Adipocyte complement related factor 30, FATP-1: Fatty acid transport protein-1.

Although established for glucose metabolism, target cells for PPAR-γ agonists and the mechanisms by which they hinder inflammation within the airways are not well defined [5]. Culminating evidence suggests that PPAR-γ may act by exerting its influence as a negative immunomodulator regulating inflammatory respiratory responses (Figure 2). Pro-inflammatory cytokines seem to be the first point of call. For example, in adipose tissue, the adipogenic action of the TZD PPAR-γ ligands are opposed by several pro-inflammatory cytokines, including tumour necrosis factor (TNF)-α and interferon (IFN)-γ (Figure 2). *In vitro*, the TZDs blocked the effects of TNF-α on both adipogenesis and insulin sensitivity and, similarly, 15D-PGJ_2 _was found to prevent IFN-γ-induced murine macrophage activation [17].

In murine macrophages and human lung epithelial cell line A549, expression of PPAR-γ was upregulated by interleukin-4 (IL-4), a cytokine critical for certain subsets of airway inflammation [17,18]. Similarly, IL-4 induced 12/15-lipoxygenase (12/15-LO), an enzyme capable of generating PPAR-γ agonists *in vivo*. 12/15-LO was also highly expressed in surface airway epithelial cells under basal conditions [17]. Nitric oxide synthases (NOS) are responsible for the *in vivo *synthesis of NO, a short-lived molecule that is an effective bactericidal agent and may also regulate expression of various pro-inflammatory genes, such as IL-8, a potent chemoattractant and activator of neutrophils. Both NOS and IL-8 play an important role in airway host defence and elevated levels of IL-8 are found in bronchoalveolar lavage fluid from intrinsic asthmatic patients [19]. The two PPAR-γ agonists, 15D-PGJ_2 _and ciglitazone dose-dependently blocked the cytokine-induced expression of the inducible form of NOS. Ciglitazone alone only slightly affected cytokine-induced IL-8 secretion, however, the agonist significantly reduced IL-8 secretion from cells pre-treated with IL-4 [17]. Therefore, PPAR-γ is expressed and upregulated by IL-4 in airway epithelial cells and through the activation of airway epithelial, PPAR-γ down-regulates expression of inflammatory mediators. In essence, PPAR-γ may act as an anti-inflammatory agent via 12/15-LO-dependent pathways [17].

Certain lung proteins may also be involved. The association of PPAR-γ with the recruitment and activation of peripheral blood monocytes, such as the potent chemokine monocyte chemoattractant protein (MCP)-1, has also been studied [20]. MCP-1 is produced by lung epithelial cells during the course of inflammatory lung diseases. Studies by Momoi's group [20] have demonstrated TZD's ability to inhibit MCP-1 protein and mRNA expression in cytokine-treated A549 lung epithelial cells.

The expression and physiological role of PPAR-γ in pulmonary nonciliated bronchiolar epithelial cells (Clara cells) and alveolar type II (AT II) epithelial cells has also been investigated [21]. These cells are highly lipogenic and are responsible for maintaining pulmonary surfactant homeostasis [22]. Among the surfactant proteins, SP-B is a 79-amino acid amphipathic peptide that is synthesised and produced in Clara cells and AT II epithelial cells. The SP-B facilitates lamellar body formation in AT II epithelial cells and phospholipid spreading during the respiratory cycles. The inhibitory effect of PPAR-γ ligands on SP-B gene expression reveals a novel mechanism in the regulation of pulmonary surfactant homeostasis [21]. In the presence of 15D-PGJ_2_, the transcriptional level of SP-B was down-regulated in respiratory epithelial cell line and whole lung explant systems. Similarly, 15D-PGJ_2 _suppressed hSP-B gene activity at the -218 to -41 promoter region in human pulmonary adenocarcinoma H441 cell line transfected with various hSP-B luciferase reporter gene constructs.

The intricate multifactorial coordination of PPAR-γ and CCAAT/enhancer-binding proteins (C/EBP) for lung development during the perinatal period has also been displayed [23,24]. C/EBPs is a family of basic leucine-zipper transcription factors controlling a wide array of genes and have been postulated to serve a central role in normal tissue development and regulation of cell proliferation or differentiation [25]. C/EBPβ and δ are known to act synergistically with PPAR-γ to promote adipocyte differentiation [23]. C/EBPα gene-deficient mice die shortly after birth due to abnormal lung histology, including interstitial thickening and hyperproliferation of AT II cells [26]. In developing foetal rat lungs, the C/EBPα, β, δ, and PPAR-γ_1 _mRNA expression was increased by 3- to 5-fold from Day 18 of gestation, peaking at 1 to 2 days before birth. However, there was a transient decline of expression during the first postnatal day and a return to prenatal levels on postnatal Day 5. In the AT II cell line, C/EBPα mRNA was not detected throughout the developmental stage; C/EBPβ and δ mRNAs expression was similar to that of whole lung, with a prenatal rise profile, whereas PPAR-γ did not display any developmental increase. The expression of PPAR-γ_2 _was not detected in whole lung or in AT II cell line [24].

Changes in the metabolism of fatty acids such as arachidonic acid may also have detrimental effects on chronic respiratory diseases including asthma, chronic bronchitis, cystic fibrosis and bronchiectasis, as well as lung injury and sepsis [27] The 85-kDa cytosolic phospholipase A_2 _(cPLA_2_) plays an essential role in the control of arachidonic acid metabolism. It has been shown that cPLA_2 _overexpression significantly increased the PPAR-γ-mediated reporter activity and this activation by cPLA_2 _may represent a novel mechanism for the control of airway inflammation [28].

### Asthma and PPAR-γ

Asthma is a widespread chronic disease, with an increasing incidence among children under 18 years of age [1]. Latest news reports headline the disease and its appearance in the elderly at an alarming rate. Sufferers are plagued with many undesirable pro-inflammatory events in the airway, including narrowing and increased production of mucous, thickening of the wall and thus reduction of the airflow through the lungs. This response is accompanied by the activation of cell types such as T cells and eosinophils and histopathological cellular airway restructuring within the airways [4,5,14]. Airway inflammation and alterations in cellular turnover are histopathologic features of asthma [4] and recently, research has disclosed the involvement of PPARs such as PPAR-γ and PPAR-α in many facets of the disease such as decreasing antigen-induced airway hyperresponsiveness, lung inflammation, eosinophilia, cytokine production and serum levels of antigen-specific IgE [29]. Airway remodelling is characterised by the increase in subepithelial membrane (SBM) and collagen deposition. A recent study displayed a positive correlation between PPAR-γ expression and SBM thickening and collagen deposition in the epithelium [4]. In the submucosa, PPAR-γ expression was related to both SBM thickening and to the number of proliferating cells. Negative correlation was found between the intensity of PPAR-γ expression in the bronchial submucosa, the airway epithelium and the smooth muscle to the forced expiratory volume (FEV_1_) values. Inhaled steroids (either administered alone or in combination with oral steroids) restrained PPAR-γ expression in all the compartments, cell proliferation, SBM thickness and collagen deposition, enhancing apoptotic death in the epithelium and the submucosa. In this study, T lymphocytes in the bronchial mucosa failed to express PPAR-γ. Therefore, PPAR-γ may be an indicator of airway inflammation and remodelling in asthma (Table 1).

**Table 1:** This table shows PPAR-γ activators, inflammatory mediators affected by PPAR-γ expression and different disorders which can be controlled by up-regulation of PPAR-γ. Abbreviations: TZDs: Thiozolidinediones, NSAIDs: Non-steroidal anti-inflammatory drugs, 15D-PGJ2: 15-deoxy-Δ^12,14^-prostaglandin J2, Cpla2: cytosolic phospholipase A_2_, IL-4: Interleukin-4, MCP:1monocyte chemoattractant protein, G-CSF: granulocyte-colony-stimulating factor, GM-CSF:granulocyte-macrophage-colony-stimulating factor, KC: keratinocyte-derived chemokine, NOS: Nitric oxide synthases, SP-B: surfactant proteins-B, MMP-9: matrix metalloproteinase 9, TGF-β: Transforming growth factor-β, IgE and IgG1: Immunoglubulin E and Immuno globulin G1, NF-κB: Nuclear factor-κB, EP2: Prostaglandin E2 receptor, PGE2: Prostaglandin E2.

**LIGANDS**	**DOWN REGULATION**	**IMPLICATION**	**UP REGULATION**	**IMPLICATION**
TZDs (Exogenous)	Cytokines (IL-8, IL-4, IL-5, IL-6 and IL-13) NOS MCP-1	Asthma and other pulmonary inflammatory diseases	aP2 UCP1 UCP3 Acrp30	Insulin resistance Obesity Hyperlipidaemia
NSAIDs (Exogenous)	SP-B AHR			
15D-PGJ2 (Endogenous)	TGF-*β* GATA-3 IgE and lgG1			
IL-4 (Endogenous)	T-cell response MMP-9 G-CSF and KC			
azelaoyl-phosphocholine (Endogenous)	GM-CSF	COPD	FATP-1 LPL (Adipose tissue)	Atherosclerosis
Eicosenoids (Endogenous)	Cyclin D1 NF-κB PGE2 EP2	Lung cancer (NSCLC, LCC)		

In ovalbumin (OVA)-sensitised BALB/c mice (a murine model of human asthma), PPAR-γ activation by ciglitazone treatment inhibited antigen-induced airway hyperresponsiveness (AHR), basement membrane thickness, collagen deposition and transforming growth factor (TGF)-β synthesis, lung inflammation, eosinophilia, cytokine production (IL-4, IL-5, IL-6 and IL-13), GATA-3 expression and serum levels of antigen-specific IgE and IgG1. *In vitro *chemotaxis and antibody-dependent cellular cytotoxicity in human or rat eosinophils were also prevented. The PPAR-γ antagonist GW9662 reversed the above effects [5,29,30]. Similarly, PPAR-γ selective agonist GI 262570 administered intranasally in OVA-induced BALB/c reduced the elevated allergen-induced bronchoalveolar lavage eosinophil and lymphocyte but not neutrophil influx. In OVA-pulsed dendritic cells (DC), rosiglitazone, a PPAR-γ agonist, averted the migration of antigen-loaded DCs in the mediastinal lymph nodes (MLN) and reduced the T-cell response in the MLNs [30]. Therefore, PPAR-γ stimulation of DCs may have a potential therapeutic role in reducing sensitisation to inhaled allergens.

In similar experiments, PPAR-γ agonist GI 262570, PPAR-α agonist GW 9578 and dual PPAR-α/γ agonist GW 2331 selectively inhibited allergen-induced bronchoalveolar lavage eosinophil and lymphocyte influx in OVA-sensitised BALB/c mice. However, PPAR-δ agonist GW 501516 had no effect. There was no inhibition of LPS-induced bronchoalveolar lavage neutrophil influx or TNF-α and keratinocyte-derived chemokine (KC) production by all agonists administered intranasally before the challenge. In A549 cells, the PPAR agonists did not inhibit intracellular adhesion molecule-1 expression. Thus, *in vitro *data suggests that PPAR effects on bronchoalveolar lavage eosinophil and lymphocyte influx may not be mediated by the antagonism of the NF-κB pathway [31].

Interleukin-5 (IL-5) is the principal regulatory cytokine mediating eosinophil airway inflammation and extending the cell's survival. Eosinophils liberate cytotoxic products at the site of inflammation, thus triggering AHR. IL-5-stimulated (but not spontaneous) eosinophil survival and eotaxin-directed chemotaxis was dose-dependently reduced by the PPAR-γ agonist troglitazone. The results indicated that upregulation of PPAR-γ in asthma may prevent further activation of pro-inflammatory cells of the airway [14].

Enzymes may also play a part in the PPAR-γ puzzle. Matrix metalloproteinase (MMP)-9 (gelatinase B) is a matrix-degrading enzyme found in human normal bronchial epithelial cells and is involved in airway wall remodelling generated by inflammatory processes. Activation of PPAR-γ by rosiglitazone or pioglitazone in human bronchial epithelial NL20 and BEAS cell lines dose-dependently limited the expression of MMP-9 gelatinolytic activity induced by TNF-α and phorbol myristate acetate. In contrast, the expression of the local inhibitor of MMP-9, tissue inhibitor type 1, was retained. In this study, however, transient transfection and electromobility shift assays **affirmed **inhibition of nuclear factor (NF)-κB activation by PPAR-γ agonists, resulting in decreased MMP-9 mRNA expression [15]. In untreated atopic asthmatic patients, there was an enhanced expression of PPAR-γ, which suggested signs of airway transformation, including increased density of the SBM and collagen deposition in the epithelium, with no relation to proliferation or apoptosis. In contrast, PPAR-γ-expressing cells in the submucosa were related to both SBM thickening and to the number of Ki67-, but not caspase-3-expressing-, cells. It was proposed that PPAR-γ might not be involved in epithelial cell turnover, but rather may manipulate extracellular matrix accumulation and submucosal cell proliferation [4] (Table 1).

PPAR-γ activation also influences lung survival factors and apotosis. In male BALB/c mice, the initial levels of the cytokines were not affected by the PPAR agonists, rosiglitazone or SB 219994. Aerosolised lipopolysaccharide (LPS) exposure caused a significant increase in neutrophil numbers in both lung lavage and tissue, however, lymphomononuclear (LMN) cell numbers in BAL fluid and lung tissue did not change. On pre-treatment with the PPAR ligands, the increase in pro-inflammatory cytokines granulocyte-colony-stimulating factor (G-CSF) and KC levels was reduced in the lung tissue but not in the lung lavage fluid. At the trial doses, the PPAR-γ agonists did not affect LMN cells numbers in the BAL nor lavage or lung tissue homogenate MMP-9 content. Rosiglitazone, when administered after the LPS insult, reduced the lung tissue G-CSF and neutrophilia levels and had no effect on KC or granulocyte-macrophage (GM-CSF) levels. The results suggested therapeutic similarities between rosiglitazone and the steroid, dexamethasone [2] (Table 1).

AMs are phagocytes involved in the ingestion and degradation of inhaled particles. This activates a variety of inflammatory processes involving enhancement of their cytotoxic capabilities. LPS-induced human AMs treated with 15D-PGJ_2 _and troglitazone showed a significant reduction of the TNF-α cytokine production. This was coupled with an increase in the expression of the scavenger receptor CD36 (which contains a functional PPAR-γ responsive element) and subsequent augmented apoptotic neutrophil phagocytosis in the ligand-treated AMs [3]. Therefore, administration of PPAR-γ synthetic agonists such as TZDs may contribute as adjunct therapeutic agents for airway diseases of the lung, such as asthma [7,14] (Table 1).

### Lung Cancer and PPAR-γ

Lung cancer is the leading cause of cancer-related death in developed countries and currently eludes the available therapies. Consequently, the prognosis of patients with lung cancer is generally poor, with a 10–15% 5 year survival rate [32]. High PPAR-γ expression has been suggested as a potential marker for lung cancer and the degree of PPAR-γ protein appears to correlate with the maturational stage, differentiated phenotype, as well as the tumour histological type and grade in lung adenocarcinoma [33,34]. Studies have indicated that upon addition of PPAR-γ selective agonists, growth of lung cancer cells was prevented through the induction of differentiation and apoptosis [35-38]. Additionally, decreased PPAR-γ expression has been correlated with poor prognosis in patients with lung cancer, suggesting that the gene expression may be further diminished as lung cancer progresses [33]. PPAR-γ-selective agonists such as ciglitazone and 15D-PGJ_2 _have diminished the growth of non-small cell lung cancer (NSCLC) cells through the induction of apoptosis, promotion of differentiation and the down-regulation of cell cycle proteins such as Cyclin D1 [35,37]. Treatment with troglitazone and pioglitazone significantly reduced the number of lung metastases and restricted NSCLC tumour progression *in vivo *[34]. Similarly, combination of ciglitizone with trichostatin (an inhibitor of histone deacetylase) demonstrated potent growth-inhibitory and differentiation-inducing activity in NSCLC, prompting the possibility of combinational differentiation therapy for the treatment of lung adenocarcinomas [37]. Likewise, untreated large cell carcinoma (LCC) cells displayed increased NF-κB activity, a pro-survival mechanism for this cancer in preventing apoptosis. Upon treatment with thalidomide, the elevated level of NF-κB activity was constrained in the presence of thalidomide and the PPAR-γ protein expression in LCC was dose-dependently increased [32]. Therefore, as activation of PPAR-γ impedes lung tumour progression, it is feasible that TZDs may serve as potential therapeutic agents for both NSCLC and LCC (Table 1).

Another aspect of carcinogenesis is the role of the inducible enzyme, cyclooxygenase (COX)-2. COX-derived prostaglandins (PG) exhibit modulation of cell proliferation, apoptosis, angiogenesis and immunity [39]. Prostaglandin E_2 _(PGE_2_) is a major COX-2 metabolite and plays an important role in tumour biology and its function is mediated through G protein-coupled PGE receptor (EP) [40]. The NSCLC cell expressing EP2 receptors, a key modulator of tumor development, has its mRNA and protein expression significantly attenuated in the presence of PPAR-γ ligands, GW1929, 15D-PGJ_2_, ciglitazone, troglitazone and rosiglitazone [41]. The effects of non-steroidal anti-inflammatory drugs (NSAIDs) on decreased lung cancer cell growth have also been examined [42,43]. Sulindac sulfide, a COX inhibitor, activated PPAR-γ at higher concentration (50 μM). Together with ciglitazone, sulindac sulfide potently suppressed NSCLC cell growth [42]. Another COX-2 inhibitor, nimesulide (which is known to induce PPAR-γ expression), has also had some success in curbing tumour growth in female nu/nu mice xenografted with subcutaneous A549 lung tumour cell line and significantly reduced intratumour PGE_2 _levels [43]. Therefore, the potential therapeutic application of NSAIDs and TZDs in the treatment and/or prevention of lung cancer are promising, however more research is still needed in order to evaluate the long-term safety and efficacy of combined NSAIDs and TZDs in lung cancer [44] (Table 1).

On the contrary, PPAR-α was not expressed in human lung cancer cell lines and, thus, respective agonists such as bezafibrate and prostanoids (PGE_2 _and PGF_2α_) did not inhibit growth of the cancer cell lines by inducing apoptosis [35].

### Other Respiratory Disorders and PPAR-γ

Cystic fibrosis is a genetic disorder characterised by functional deficiencies of the reproductive, digestive and respiratory systems. With the help of genetic mapping and improved, more consistent treatment, patients are enjoying longer and fulfilled lives. Adding to the improved outlook, it is believed that respiratory PPAR-γ expression is altered in tissues deficient in the normal cystic fibrosis transmembrane regulator protein (CFTR). It was found that PPAR-γ expression was decreased significantly in (CFTR)-regulated tissues (colon, ileum and lung) from exon 10 CFTR (cftr^_/_^) mice compared to wild-type mice. In contrast, no differences were found in fat and liver. In the lung tissue of both mice types, there was a mixed labelling of both nuclei and cytoplasm localised to larger bronchi and a diffuse lighter staining of the remaining tissue [45].

The deficiency of GM-CSF is strongly implicated in the pathogenesis of pulmonary alveolar proteinosis (PAP), a rare interstitial lung disease manifested by surfactant accumulation in alveolar airspaces. In PAP individuals, both PPAR-γ mRNA and the PPAR-γ-regulated lipid scavenger receptor, CD36 were reduced in AMs when compared to healthy subjects. PPAR-γ and CD36 deficiency in PAP was cell type-specific in the lung (i.e. found in AM and not in bronchial epithelial cells). *In vitro *and *in vivo *GM-CSF treatment of PAP patients fully restored PPAR-γ to healthy control levels [46].

As for asthma patients, cell-proliferating lesions obstruct the vessel lumen and promote pulmonary arterial pressure and reduced blood flow in COPD patients [16,47] (Table 1). In asthma, the eosinophil survival indicator, GM-CSF, is prominent in bronchoalveolar lavage fluid, serum and lung tissue. On the contrary, COPD is characterised by neutrophilia [48]. It has been confirmed that both GM-CSF and the related survival factor, G-CSF are involved in the survival of the neutrophils. Consequently, these factors may aggravate and extend the inflammatory response in neutrophil-related inflammatory lung diseases such as COPD [49,50].

Activation of PPAR-γ by 15D-PGJ_2 _and ciglitazone induced apoptosis and impeded serum-induced cell growth more effectively than the steroid dexamethasone in HASM. Moreover, PPAR-γ ligands and dexamethasone hampered the IL-1β-induced release of GM-CSF. However, PPAR-γ ligands, but not dexamethasone, similarly deterred G-CSF release. The above actions of 15D-PGJ_2 _were not dependent on the activation of a traditional cell surface prostanoid receptor. Agents that obstruct proliferation of HASM cells, as well as CSF release, would represent potential new therapies to treat COPD and steroid-insensitive asthma [16] (Table 1).

## Conclusion

It appears that chronic lung disorders are not confined to a particular race, sex or age. Studies delving into respiratory diseases have reached a crucial point and the increasing incidence and potential fatality of these debilitating diseases has emphasised the urgent quest for novel therapeutic avenues vital to the control and ultimate elimination of such disease. The role of PPAR-γ in regulating adipocyte differentiation and glucose homeostasis has been established and, consequently, further research has uncovered its involvement in inflammatory events of cardiac and, more recently, airway diseases. Antagonism of the pro-inflammatory pathways in respiratory diseases is the likely mechanism of action of the PPARs and their respective agonists. Research on the physiological role of PPAR-γ in the lung is still in its infancy, however, continued advancement in this field will unravel the co-existence and interactions of the PPAR-γ gene and related ligands such as 15D-PGJ_2 _and TZDs in the prevention or treatment of inflammatory respiratory diseases. It is unlikely that the current PPAR-γ agonists will be used as a monotherapy in airway diseases such as asthma and lung cancer. However, with improved comprehension of the full biological and physiological role of PPAR-γ in these diseases, novel and more potent agonists could be designed to include effective administration of anti-inflammatory therapies with minimal side effects. This could also extend to tackling more elusive or less common lung disorders such as cystic fibrosis, PAP and COPD.

It is unanimously agreed that the PPAR-γ anti-inflammatory pathways must be correctly identified for the particular disease state, as this will have important implications for the type of treatment and its effective administration. This would be determined by factors such as the receptor's presence in the particular sections of the lung (lung tissue compartment versus airway lumen), its expression in specific lung cell types and its influence on pro-inflammatory cytokines, enzymes, proteins, fatty acid metabolism and subsequent pathways. Therefore, it is anticipated that PPAR-γ expression will become a potential indicator of many airway inflammatory diseases leading to a possible prevention or treatment therapeutic application.

## Abbreviations

Peroxisome proliferator-activated receptors (PPAR); 15-deoxy-Δ^12,14^-prostaglandin J2 (15D-PGJ_2_); thiazolidinediones (TZD); chronic obstructive pulmonary disease (COPD); PPAR-response element (PPRE); direct repeat (DR); 9-cis-retinoic acid receptors (RXR); alveolar macrophages (AM); human airway smooth muscle (HASM); tumour necrosis factor (TNF); interferon (IFN); interleukin-4 (IL-4); 12/15-lipoxygenase (12/15-LO); nitric oxide synthases (NOS); monocyte chemoattractant protein (MCP); alveolar type II (AT II); surfactant protein, (SP); CCAAT/enhancer-binding proteins (C/EBP); cytosolic phospholipase A_2 _(cPLA_2_); subepithelial membrane (SBM); forced expiratory volume (FEV_1_); ovalbumin (OVA); antigen-induced airway hyperresponsiveness (AHR); transforming growth factor (TGF); Immunoglubulin E and Immunoglobulin G1 (IgE and IgG1); dendritic cells (DC); mediastinal lymph nodes (MLN); interleukin-5 (IL-5); matrix metalloproteinase (MMP); nuclear factor (NF); lipopolysaccharide (LPS); lymphomononuclear (LMN); granulocyte-colony-stimulating factor (G-CSF); keratinocyte-derived chemokine (KC); non-small cell lung cancer (NSCLC); large cell carcinoma (LCC); cyclooxygenase (COX); prostaglandin E_2 _(PGE_2_); G protein-coupled PGE receptor (EP); non-steroidal anti-inflammatory drugs (NSAIDs); ystic fibrosis transmembrane regulator protein (CFTR); pulmonary alveolar proteinosis (PAP)
